# Supplementary material for: Spatiotemporal dynamics of information encoding revealed in orbitofrontal high-gamma
Source: Nat Commun. 2017 Oct 26;8:1139. doi: 10.1038/s41467-017-01253-5 (PMC5658402; doi:10.1038/s41467-017-01253-5)
Supplement: Supplementary file 1 — Supplementary Information [file 41467_2017_1253_MOESM1_ESM.pdf]

Supplementary Information

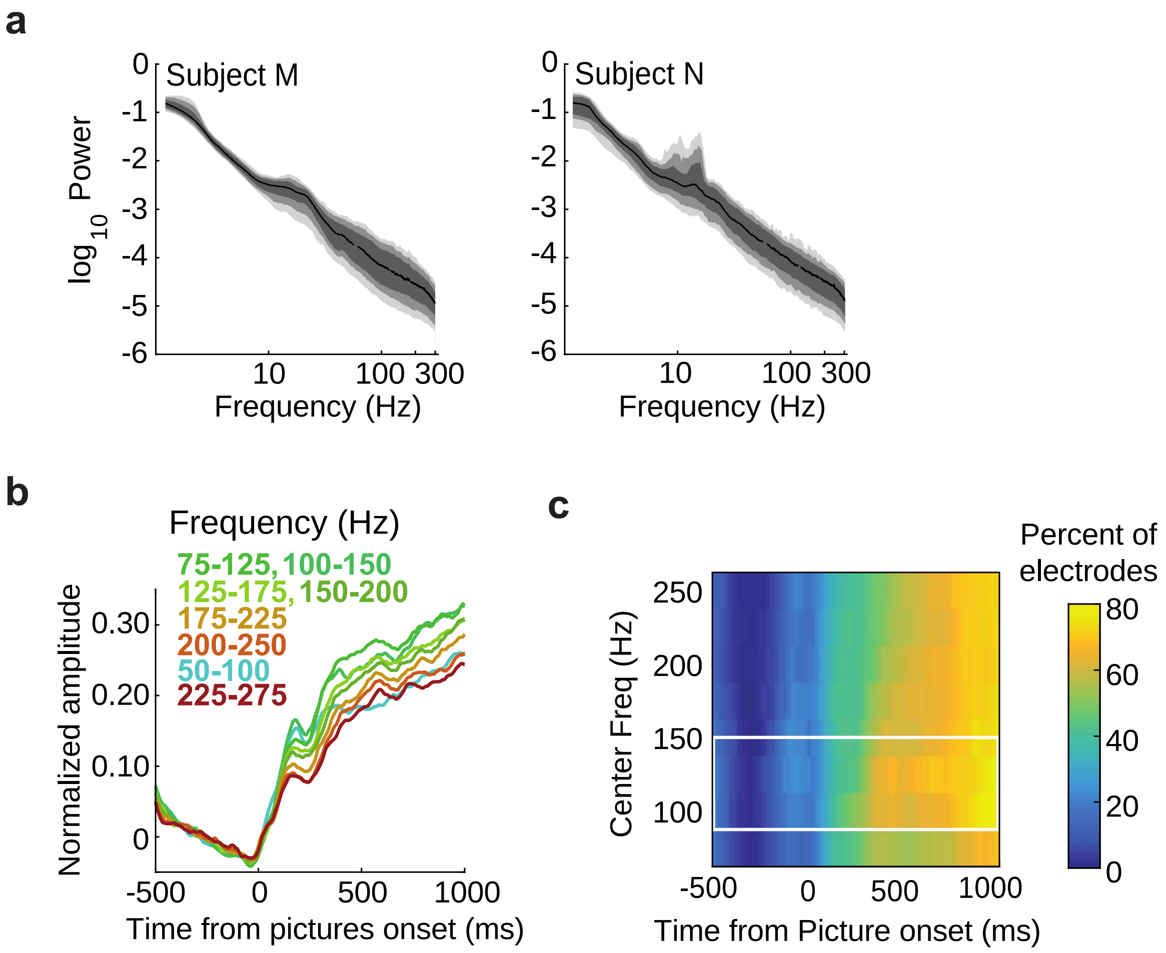

Supplementary Figure 1. To determine the optimal frequency range above 50 Hz for extracting task-relevant information, we first assessed the average power spectra to determine whether any frequencies had high power suggestive of an oscillatory band or dominant range. **(a)** In both subjects, log-log plots of power across frequencies showed a peak around 10 Hz, but were approximately linear in high frequencies, with no narrow band peaks, indicative of a power law relationship, as expected in a broadband signal. The line shows the median power across electrodes, shaded areas show the 5th – 95th percentile measures (outermost), 10th – 90th percentile (middle) and 20th – 80th percentile (innermost) for each subject. **(b)** Since there was no evidence of a dominant high frequency band, we aimed to find a range with high signal to noise in this setting. We divided the signal into 8 frequency bands with 50 Hz bandwithds overlapping by 25 Hz, from 50 to 275 Hz. Analytic amplitudes were extracted for each band and aligned to the appearance of the reward-predicting picture. Signal amplitudes increased following picture onset, and this was highest in the range of 75 - 150 Hz. All responses were normalized to the pre-stimulus period within the band, so these results cannot be attributed to lower power in higher frequencies. Instead, they likely reflect a reduction in the signal relative to noise as frequency increased. Note that the decrease in signal amplitude prior to picture onset resulted from a decrease over time following the reward epoch on the previous trial. **(c)** To further quantify this, t-tests compared the signal amplitude in each band to the amplitude during a 450 ms fixation epoch before picture onset, on a channel-by-channel basis. Many electrodes had significantly elevated responses ($p \leq 0.01$ uncorrected), but the highest prevalence was in the range of 75-150 Hz, where 70-80% of electrodes responded above pre-stimulus baseline. Based on these results, we focused further analyses on 75-150 Hz (white box).

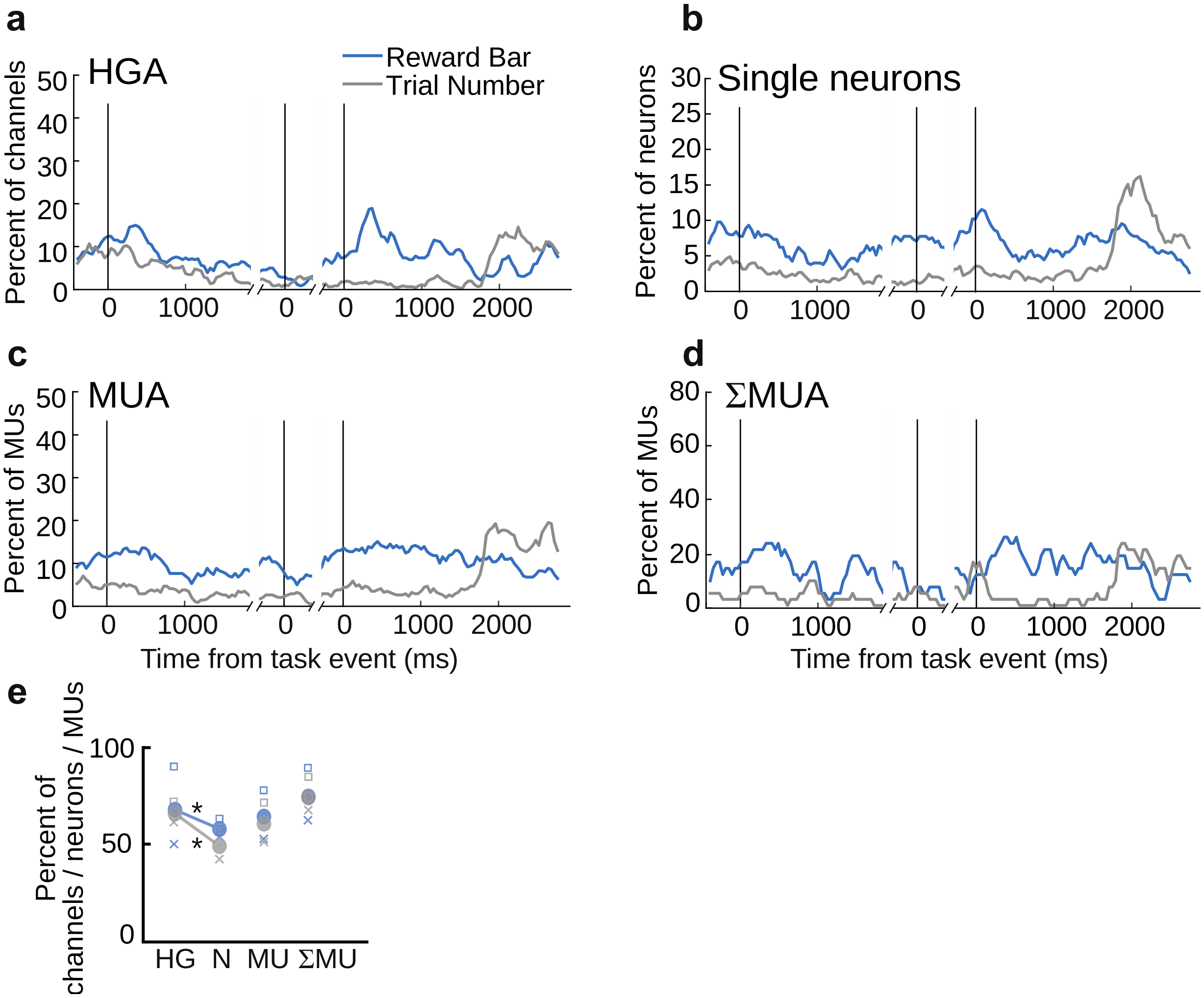

Supplementary Figure 2. At any given time, a small but consistent percentage of HGA channels **(a)**, neurons **(b)**, MUA channels **(c)**, or ΣMUA signals **(d)** encoded the size of the reward bar (blue) or the trial number within a 4-trial block (gray). However, these were not the same signals across the entire trial. **(e)** Cumulatively 50% or more of each signal encoded bar size or trial number at some time point. There was significantly more encoding of both variables in HGA than neurons (χ^2 tests, * $p < 0.001$). However, in all signals, reward bar size and trial number were intermittently encoded across many channels in OFC. This unusual pattern is unlikely to be false discoveries, as we had a nearly zero false discovery rate for picture-related variables, described above, preceding the appearance of the picture.

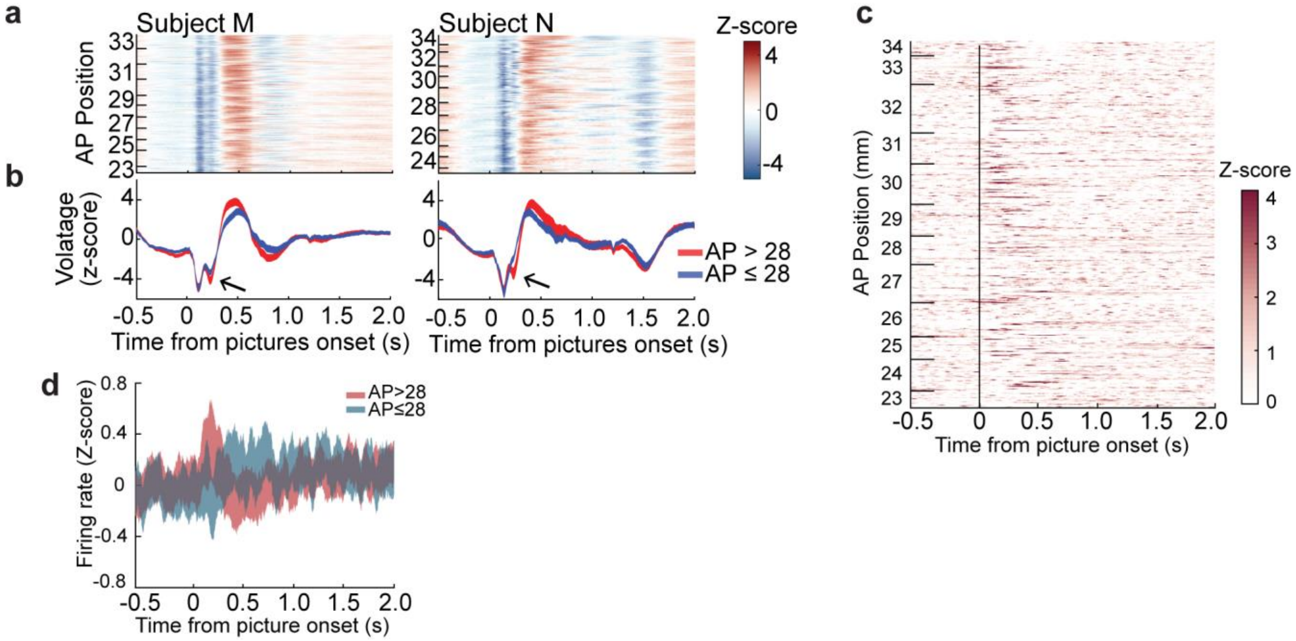

Supplementary Figure 3. Broadband ERPs. **(a)** The LFP on each electrode was assessed without applying a bandpass filter by aligning each trial to picture onset and sorting the channels by AP position. **(b)** ERPs were averaged across anterior (red) and posterior (blue) electrodes (shading shows 99% CI). ERP shape varied slightly between subjects, so each is shown separately. The arrows indicate a negative voltage deflection occurring around 230 ms that aligned temporally with the HGA peak in anterior electrodes. **(c)** Firing rates of all neurons (z-scored), averaged across trials, aligned to picture onset, and sorted by AP position as shown in **a**. **(d)** Firing rates as shown in **c**, averaged across all anterior (red) or posterior (blue) sites. There was a slight, but less pronounced, peak in firing rates among anterior neurons, that co-occurred with the negative ERP deflection, and a delayed peak among posterior neurons.

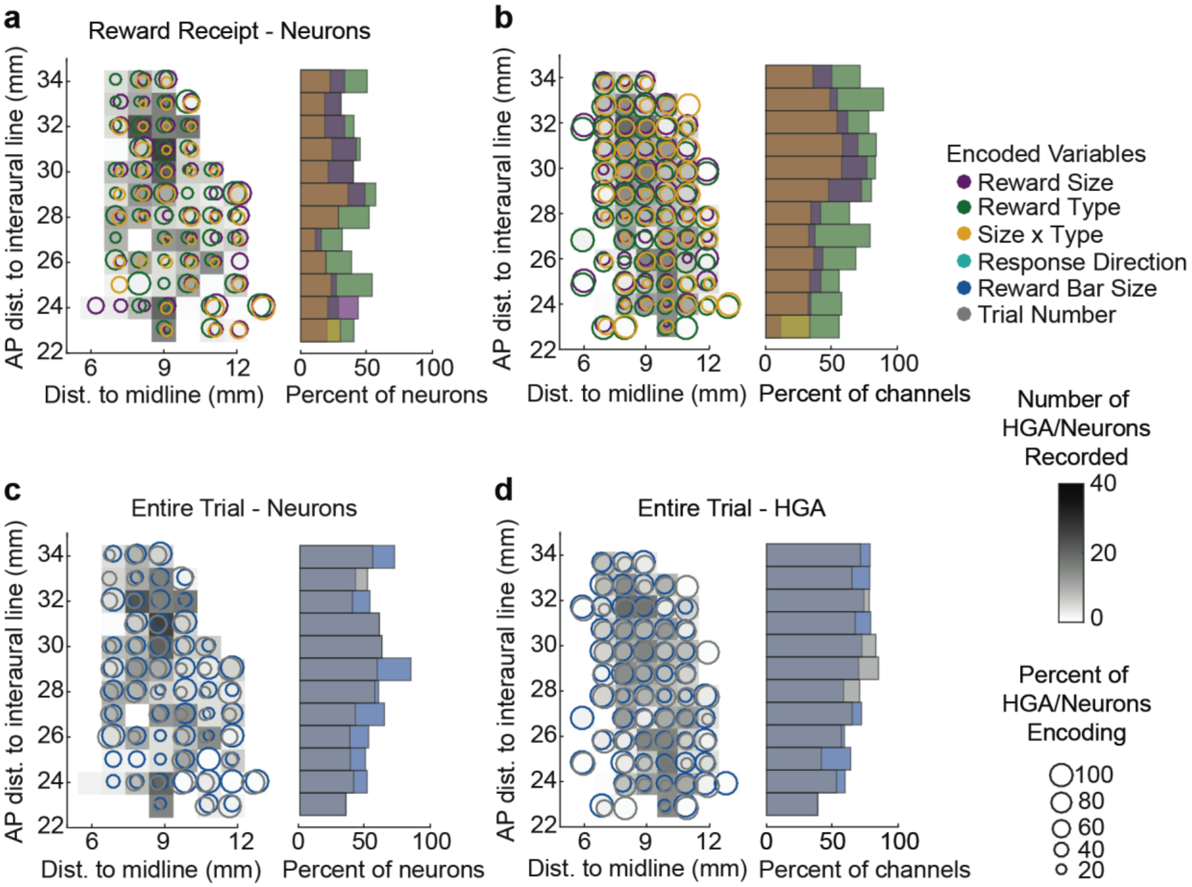

Supplementary Figure 4. We performed the same analyses shown in Figure 5a-b (in the main manuscript) on task variables related to reward receipt **(a-b)**, and the reward bar length and trial number within a block, the two variables that were constant across the entire trial **(c-d)**. Among neurons, only trial number encoding showed a weak tendency to be represented in anterior OFC (χ^2_{1df} = 9.1, corrected p = 0.02), but this only reached significance in one subject (subject M χ^2_{1df} = 0.88, corrected p > 0.9, subject N χ^2_{1df} = 8.2, corrected p = 0.04). In HG, this effect was also present and stronger (χ^2_{1df} = 23.6, corrected p = 1.1×10^{-5}), but was only significant in one subject (subject M χ^2_{1df} = 27.9, corrected p = 1.2×10^{-6}, subject N χ^2_{1df} = 1.8, corrected p > 0.9). Similarly, there were discrepancies between subjects in HG encoding of the other post-reward variables, such that subject M consistently had more encoding in anterior OFC of received reward size, type and size x type interaction (χ^2_{1df} > 16, corrected p < 0.0006), and subject N showed no differences (χ^2_{1df} < 3, corrected p > 0.8). Among neurons, no anatomical trends in post-reward variables were found in either subject (χ^2_{1df} < 7, corrected p < 0.08). Overall, inter-subject variability obscured clear trends in post-reward and entire trial variables, and in general these variables tend to be represented throughout OFC.

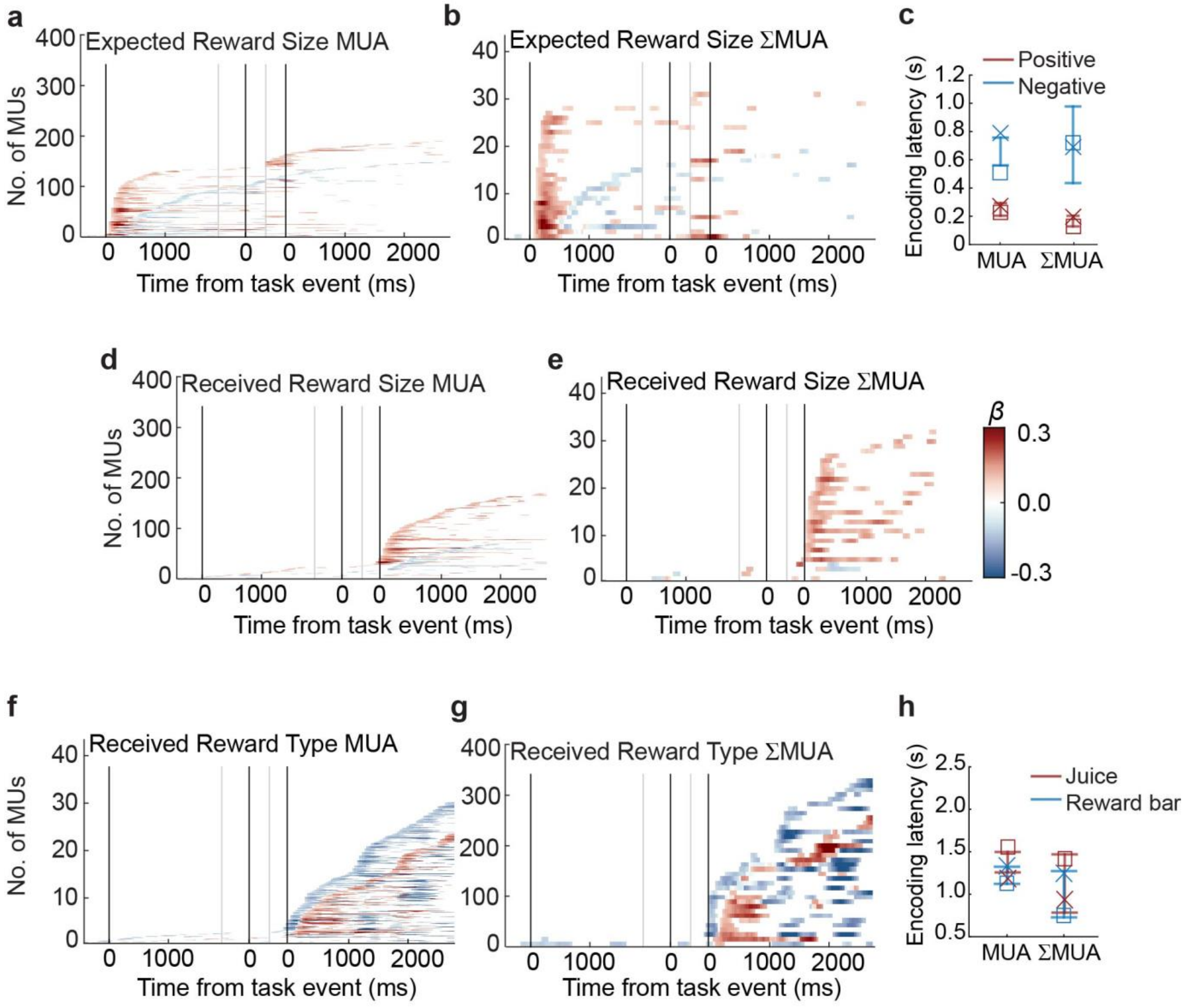

Supplementary Figure 5. Valence encoding in MUA and ΣMUA. MUA **(a)** and ΣMUA **(b)** encoding of expected reward size, separate by valence. **(c)** Both signals showed a latency difference for positive and negative encoding similar in to HGA (see Figure 6 b & d in the main text). **(d)** MUA encoding of received reward size was intermediate to single neurons and HGA, whereas **(e)** ΣMUA encoding of received reward size was similar to HGA (see Figure 8 in the main text). **(f)** MUA and (g) ΣMUA encoding of recieved reward type were both more similar to single neurons, and lacked the overwhelming response to juice seen in HGA. **(h)** Mean encoding latencies were similar for both outcomes, as in single neurons. Error bars = 95% CI, x = subject M, square = subject N.

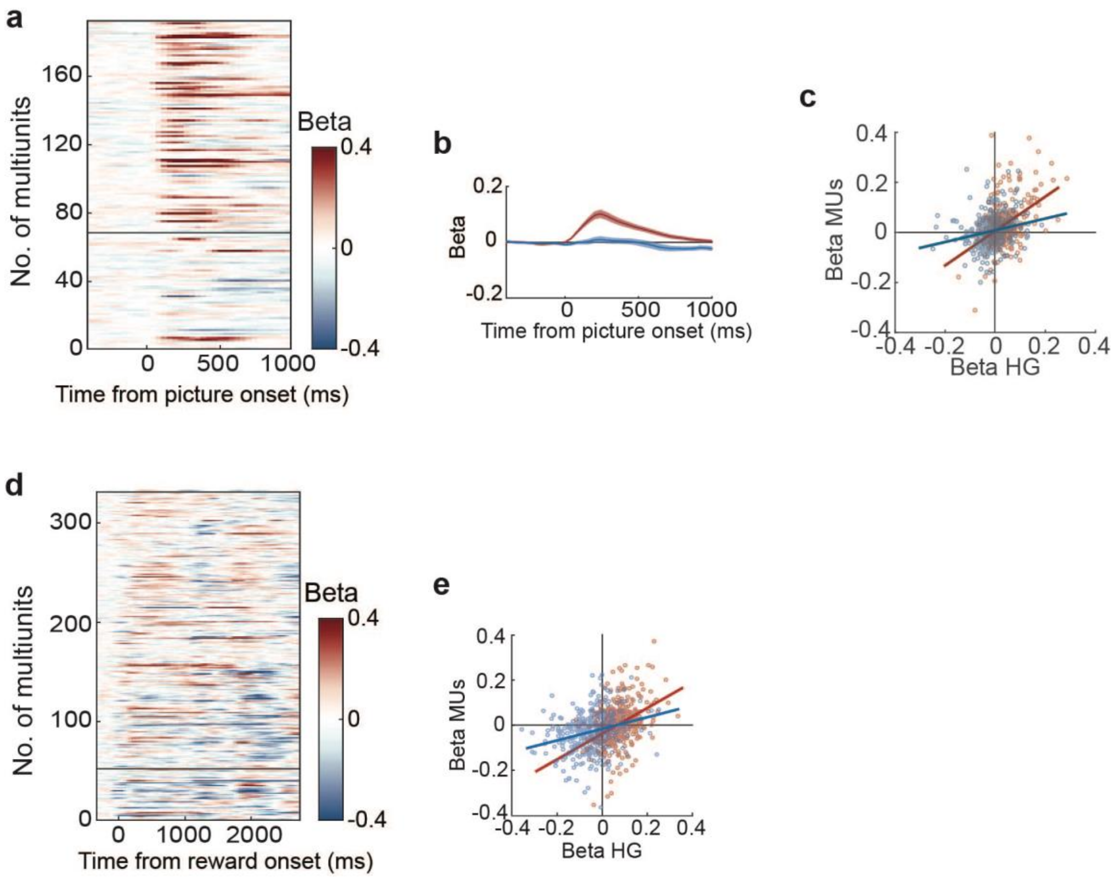

Supplementary Figure 6. Our results suggest only a weak relationship between single neuron activity and HGA, however, we believe this is because signals from individual neurons are aggregated across heterogeneous pools to give rise to HGA. If this were true, other aggregate signals may resemble HGA more closely. By combining the firing of multiple neurons, MUA aggregates firing and also captures the synchronicity of local neurons. **(a)** We identified the HGA channels with significant positive or negative encoding of picture value within the first 1 s after the stimulus appeared, as well as the MUA recorded on the same electrode (n = 166 pairs, 108 positive, 58 negative), and assessed all beta coefficients for these pairs. Compared to single neurons-HGA pairs in Figure 7 of the main text, the beta coefficients for MUA followed an intermediate pattern. That is, on electrodes with positive or negative HGA encoding (above and below the black line respectively), there was a tendency for MUA to have corresponding positive or negative beta coefficients. A Chi-square test comparing the signs of beta in HGA versus MUA showed a stronger effect than that between HGA and neurons (χ^2_{1df} = 19.6, p = 9.6 x 10^{-6}). **(b)** The average beta coefficients on channels with positive (red) and negative (blue) HGA encoding patterns were more distinct than single neurons, but weaker than HGA. **(c)** Pearson correlation of average beta coefficients between all HGA-MUA pairs showed a positive relationship in two 500 ms epochs after stimulus onset (R = 0.45, 0.21 and p = 4.4 x 10-19, 4.3 x 10-5, red and blue respectively), and the population of coefficient values shifted from having more positive betas in the first 500 ms and more negative betas in the second 500 ms. This is similar to what was observed in single neurons, though the relationships between MUA and HGA were slightly stronger. **(d)** As in panel **a**, we identified HGA channels with significant encoding of received reward type, and plotted the beta coefficients for reward type from MUA activity on the same electrodes (juice-preferring and secondary-preferring above and below the line respectively). The pattern in MUA activity was more varied than that in HG (see Figure 6d in the main text). **(e)** However, correlation of the average beta coefficients across all HGA-MUA pairs found significant correlations in the first 500 ms after reward onset (red, R = 0.39, p = 1.6 x 10-14), and in the a later epoch 2500 – 3000 ms after reward onset (blue, R = 0.26, p = 7.5 x 10-7), although the population of beta coefficients tended to shift from initially positive (juice-preferring) to negative (secondary-preferring).
